# An ultrasound-based radiomics model to distinguish between sclerosing adenosis and invasive ductal carcinoma

**DOI:** 10.3389/fonc.2023.1090617

**Published:** 2023-03-07

**Authors:** Qun Huang, Wanxian Nong, Xiaozhen Tang, Yong Gao

**Affiliations:** Department of Ultrasound, First Affiliated Hospital of Guangxi Medical University, Nanning, Guangxi, China

**Keywords:** sclerosing adenosis, invasive ductal carcinoma, ultrasound, radiomics, model

## Abstract

**Objectives:**

We aimed to develop an ultrasound-based radiomics model to distinguish between sclerosing adenosis (SA) and invasive ductal carcinoma (IDC) to avoid misdiagnosis and unnecessary biopsies.

**Methods:**

From January 2020 to March 2022, 345 cases of SA or IDC that were pathologically confirmed were included in the study. All participants underwent pre-surgical ultrasound (US), from which clinical information and ultrasound images were collected. The patients from the study population were randomly divided into a training cohort (n = 208) and a validation cohort (n = 137). The US images were imported into MaZda software (Version 4.2.6.0) to delineate the region of interest (ROI) and extract features. Intragroup correlation coefficient (ICC) was used to evaluate the consistency of the extracted features. The least absolute shrinkage and selection operator (LASSO) logistic regression and cross-validation were performed to obtain the radiomics score of the features. Based on univariate and multivariate logistic regression analyses, a model was developed. 56 cases from April 2022 to December 2022 were included for independent validation of the model. The diagnostic performance of the model and the radiomics scores were evaluated by performing the receiver operating characteristic (ROC) analysis. The calibration curve and decision curve analysis (DCA) were used for calibration and evaluation. Leave-One-Out Cross-Validation (LOOCV) was used for the stability of the model.

**Results:**

Three predictors were selected to develop the model, including radiomics score, palpable mass and BI-RADS. In the training cohort, validation cohort and independent validation cohort, AUC of the model and radiomics score were 0.978 and 0.907, 0.946 and 0.886, 0.951 and 0.779, respectively. The model showed a statistically significant difference compared with the radiomics score (*p*<0.05). The Kappa value of the model was 0.79 based on LOOCV. The Brier score, calibration curve, and DCA showed the model had a good calibration and clinical usefulness.

**Conclusions:**

The model based on radiomics, ultrasonic features, and clinical manifestations can be used to distinguish SA from IDC, which showed good stability and diagnostic performance. The model can be considered a potential candidate diagnostic tool for breast lesions and can contribute to effective clinical diagnosis.

## Introduction

Sclerosing adenosis (SA) is a common benign lesion that may mimic breast malignancy clinically, radiologically, and pathologically (1–4). SA is usually asymptomatic or palpated with a mass, which is unexpectedly found in premenopausal women who have been examined using imaging or histopathology for other reasons (2). SA is often radiologically evaluated as a malignancy. Pathologically, SA is a complex proliferative change consisting of enlarged and twisted nodules and containing repeated and crowded acini accompanied by significant myoepithelial and interstitial fibrosis (5). SA often imitates malignancy, leading to misdiagnosis and excessive biopsies, which have a negative influence on women’s physical and mental health. As the most common breast cancer, IDC may coexist with SA, making it difficult to distinguish between them (6). However, surgical resection is the main treatment for IDC due to its invasiveness and metastasis, whereas follow-up procedures are performed for SA (7).

The conventional breast ultrasound (US) plays a key role in screening, diagnostic imaging, and interventional breast surgery for breast lesions. For patients, US is relatively quicker, more comfortable, less expensive, and radiation-free. The American College of Radiology Breast Imaging Report and Data System (ACR BI-RADS) has developed a standardized vocabulary to describe the findings of US examinations, and has established a system to classify these findings and the probability of malignant tumors (8, 9). However, US and BI-RADS both depend on the subjective observations of radiologists. Therefore, exploring the use of a non-invasive and objective method to differentiate between benign and malignant lesions is crucial.

Texture analysis technology extracts texture feature parameters by certain image processing systems, which can objectively and quantitatively provide information about the lesions that cannot be identified by the naked eye (10, 11). MaZda is a software package used for 2D and 3D image texture analyses, and it provides a complete path for the quantitative analysis of image textures. It is effective in its use for various imaging analyses, including X-rays, US, and magnetic resonance imaging. It has been proven to be an efficient and reliable tool for quantitative image analyses, providing more accurate and objective medical diagnoses (12–15).

A logistic regression model is based on a multivariate regression analysis, integrating multiple predictors and using multiple indicators to diagnose or predict the occurrence or progress of diseases (16, 17). To our knowledge, there is no model based on an ultrasonic texture analysis used to distinguish between SA and IDC. We aimed to develop and validate an ultrasound-based radiomics model to differentiate between SA and IDC, which could be a potential candidate diagnostic tool for breast lesions and could help to avoid misdiagnosis and unnecessary biopsies.

## Materials and methods

### Study population

This retrospective study was approved by the Research Ethics Committee of the First Affiliated Hospital of Guangxi Medical University. We retrospectively reviewed the medical records of 345 consecutive female patients (345 lesions) in our hospital from January 2020 to March 2022, including 76 cases of SA and 269 cases of IDC. Patients from the study population were randomly divided into a training cohort (n=208, mean age: 51.3 ± 12.2 years) and a validation cohort (n=137, mean age: 51.5 ± 10.2 years). The consistency between the two cohorts was tested. In addition, patients from our hospital from April 2022 to December 2022, including 26 cases of SA and 30 cases of IDC, were included for independent validation (n = 56, mean age: 48.3 ± 13.6 years).

The inclusion criteria were as follows: (1) a breast US was performed before biopsy or surgery; (2) US images were available for qualitative and radiomic analysis; (3) all participants were confirmed as SA or IDC by biopsy or surgical pathology; (4) all patients had not received systemic hormone therapy or neoadjuvant chemotherapy; (5) the clinical information and US images were complete; and (6) only a lesion in the largest or highest BI-RADS category was included for patients with multiple lesions.

The exclusion criteria were as follows: (1) the poor quality of ultrasonic images affected the texture analysis; (2) the pathological result was indefinite; (3) patients had received systemic hormone therapy or neoadjuvant chemotherapy; (4) clinical information and US images were lacking; and (5) the lesion was too large to delineate the ROI.

**Figure 1:**
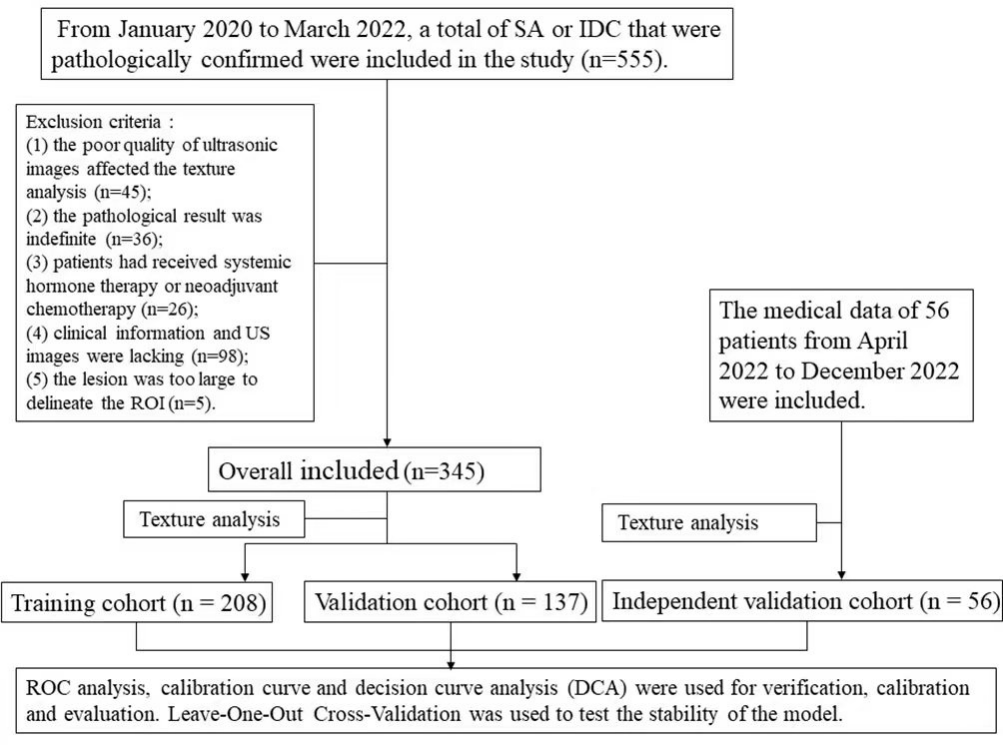
The flow chart of the study.

The flow chart of the study was shown in **Figure 1**.

### Breast ultrasound technology

All patients underwent a pre-surgical US examination. The patients were in a supine position with their hands raised above their heads to fully expose the breast. Color Doppler ultrasound instruments included GE LOGIQ E9, VOLUSON E9 (General Electric Company, Boston, USA), or HITACHI ARIEETTA 70 (HITACHI Ltd., Tokyo, Japan) with a linear array probe and a frequency of 9-12 MHz.

The standard store images of breast lesion included at least two vertical sections, one of which showing the maximum diameter of the lesion. The images with the clearest and most complete demonstration of lesions were chosen. The focus was located slightly below the lesion, and the frequency range was 9-12MHz. Each lesion was classified into a category (3, 4A, 4B, 4C, or 5) according to the 5th edition of ACR BI-RADS US. According to ACR BI-RADS classification, BI-RADS 4A means that the degree of malignancy is very low, and the possibility of benign lesions is far greater than that of malignant lesions. According to relevant literature, lesions of BI-RADS 3 or 4A were considered to be negative, and lesions of BI-RADS 4B, 4C or 5 were considered to be malignant in our study (18). The ultrasonic features of the breast lesions were recorded, including maximum size, shape, echo pattern, echo distribution, boundary, orientation, posterior feature, calcification, vascularity distribution, and associated features. All lesions were examined and evaluated by two ultrasound doctors with more than five years of experience with breast US. In the case of a disagreement, a final consensus was reached through a discussion.

The maximum size was the largest diameter of the tumor. The shape was defined as regular or irregular. The echo pattern was divided into hypoechoic, or complex echo. The echo distribution was divided into uniform or non-uniform types. The boundary was interpreted as well-circumscribed or obscure. The orientation was depicted as whether or not the breast lesion was parallel to the chest wall. The posterior acoustic features were classified as attenuated or not. The vascularity distribution was recorded as absent or internal (1). Associated features included duct ectasia, and palpable mass.

### Pathological findings

The histopathological results of all lesions were obtained from the surgical resection report. Each specimen was placed in a formalin solution, and then histopathological treatment was carried out using the standard procedures. The final pathological results were evaluated by experienced pathologists.

### Radiomic analysis

**Figure 2:**
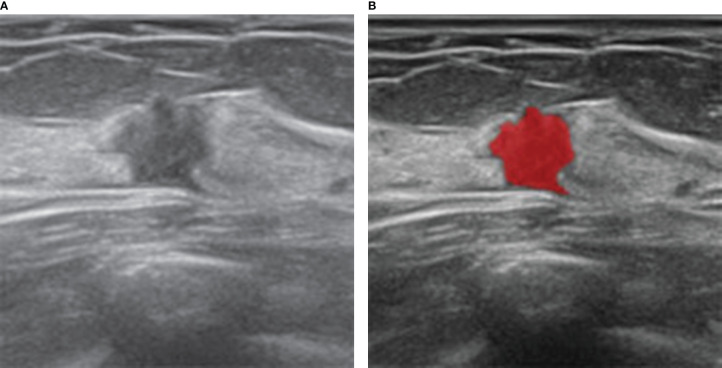
Ultrasound and histopathologic findings of a 35-year-old woman with SA. **(A)** The ultrasound image showed a hypoechoic lesion with irregular shape. The lesion was classified as BI-RADS 4C and considered malignant, which was considered benign by our model. **(B)** ROI was manually drawn in red by MaZda software along the edge of the lesion.

The section of the largest diameter of the lesion was selected to draw ROI by one ultrasound doctor with more than ten years experience of breast US. ROI was set to be 0.1-0.2cm along the inner edge of the lesion. The ultrasound gray-scale images were imported into MaZda software (Version 4.2.6.0), and the ROI results were then delineated manually (**Figure 2**). After normalization, a total of 279 descriptors were used to characterize the gray-scale image texture using MaZda software, including nine texture features based on the histogram, 11 features based on the co-occurrence matrix (derived from 20 co-occurrence matrices produced for four directions and five inter-pixel distances), five features based on the run-length matrix (each in four different directions), five features based on a gradient map, five features based on an autoregressive model, and up to 20 features based on the Haar wavelet transform (12).

In order to select the features with good reproducibility and stability to build the model, 30 ultrasound images of breast lesions were randomly selected. The ROI was drawn by another ultrasound doctor with more than ten years experience of breast US and the features were extracted again. Intragroup correlation coefficient (ICC) was used to evaluate the consistency between the ROI extraction features, which was drawn by two ultrasound doctors. The features with ICC greater than or equal to 0.75 were considered to have good reproducibility and stability. The least absolute shrinkage and selection operator (LASSO) logistic regression and cross-validation were performed to select the significant features. The selected features were used to establish the radiomics score.

### Development and validation of the model

We conducted univariate and multivariate logistic regression analyses to explore the influencing factors. The candidate factors included clinical information, ultrasonic features, BI-RADS, and the radiomics score. In the training cohort, variables selected by the univariate analysis (*p*<0.05) were used for the multivariate logistic regression to determine the independent risk factors for the model. On the basis of the validation cohort, the discrimination, calibration, and clinical usefulness of the model were evaluated. In addition, the logistic score of each patient in the independent validation cohort was calculated using our model. The ROC curves were plotted to assess the diagnostic performance of the model (19). The area under the ROC curve (AUC) was used to quantify discrimination. The calibration curve was used to examine the model’s predictive accuracy. To determine the clinical usefulness of the model, a decision curve analysis (DCA) was performed (20). Leave-One-Out Cross-Validation (LOOCV) was used to test the stability of the model, which was graded as very good (Kappa value of 0.80 to 1.00), good (Kappa value of 0.60 to 0.80), fair (Kappa value of 0.40 to 0.60), moderate (Kappa value of 0.20 to 0.40) or poor (Kappa value<0.20).

### Statistical analysis

The statistical analysis was conducted using R software (version 4.1.3) and SPSS 26.0 (Chicago, IL). For the categorical variables, the Chi-square test was used, although when necessary, Fisher’s exact test was used. The Student’s t-test was used to compare the continuous variables with a normal distribution. The reported statistical significance levels were all two-sided, and a *P* value< 0.05 was considered significant.

The “caret” package of R software was used to randomly split the total data, 60% of which was included in the training cohort and the remaining 40% in the verification cohort. At the same time, the package was also used for cross-validation. The “glmnet” package was used for the LASSO regression. The “glm” function of R software was used for the logistic regression analysis. The “Cairo” package was used to plot the model. The “pROC” package was used to plot the ROC curves and to measure the AUCs, which were compared using DeLong’s test. The “calibrate” function was used for the calibration curves. The “decision_curve” function was used to perform the DCA.

## Results

### Study population

A total of 401 lesions from 401 female patients (mean age: 50.9 ± 11.8 years, age range: 21-89 years) were recruited, including 102 SA (mean age: 47.1 ± 12.7 years, age range: 21-83 years) and 299 IDC (mean age: 52.2 ± 11.2 years, age range: 23-89 years). There were 208 patients with 208 lesions in the training cohort (mean age: 51.3 ± 12.2 years), 137 patients with 137 lesions in the validation cohort (mean age: 51.5 ± 10.2 years), and 56 patients with 56 lesions in the independent validation cohort (mean age: 48.3 ± 13.6 years).

### Clinical and ultrasonic characteristics

The clinical and ultrasonic characteristics of the training cohort and the verification cohort were shown in **Table 1**. There were no statistical differences in 14 observation indexes (*p*>0.05) between the training cohort and the verification cohort, which indicated that the consistency between the two cohorts was good.

**Table 1:** The clinical and ultrasonic characteristics in the training and validation cohorts.

	Training cohort(n=208)	Validation cohort(n=137)	*P*-value
Age (years)	51.3 ± 12.2	51.5 ± 10.2	0.863
Pathology			0.962
SA IDC	46 (22.1%) 162 (77.9%)	30 (21.9%) 107 (78.1%)	
BI-RADS			0.509
3-4A 4B-5	44 (21.2%) 164 (78.8%)	25 (18.2%) 112 (81.8%)	
Tumor Size (cm)	2.3 ± 1.3	2.3 ± 1.1	0.867
Duct Ectasia			0.981
None Ectasia	199 (95.7%) 9 (4.3%)	131 (95.6%) 6 (4.4%)	
Palpable Mass			0.217
None Palpable	36 (17.3%) 172 (82.7%)	17 (12.4%) 120 (87.6%)	
Echo Pattern			1.000
Hypoechoic Complex Echo	202 (97.1%) 6 (2.9%)	133 (97.1%) 4 (2.9%)	
Echo Distribution			0.053
Uniform Non-Uniform	21 (10.1%) 187 (89.9%)	6 (4.4%) 131 (95.6%)	
Boundary			0.630
Well-Circumscribed Obscure	92 (44.2%) 116 (55.8%)	57 (41.6%) 80 (58.4%)	
Shape			0.455
Regular Irregular	20 (9.6%) 188 (90.4%)	10 (7.3%) 127 (92.7%)	
Orientation			0.478
Parallel Not Parallel	170 (81.7%) 38 (18.3%)	116 (84.7%) 21 (15.3%)	
Posterior Feature			0.993
None Attenuation	173 (83.2%) 35 (16.8%)	114 (83.2%) 23 (16.8%)	
Calcification			0.828
None Calcification	89 (42.8%) 119 (57.2%)	57 (41.6%) 80 (58.4%)	
Vascularity Distribution			0.994
Absent Internal	76 (36.5%) 132 (63.5%)	50 (36.5%) 87(63.5%)	

### Radiomic analysis

**Figure 3:**
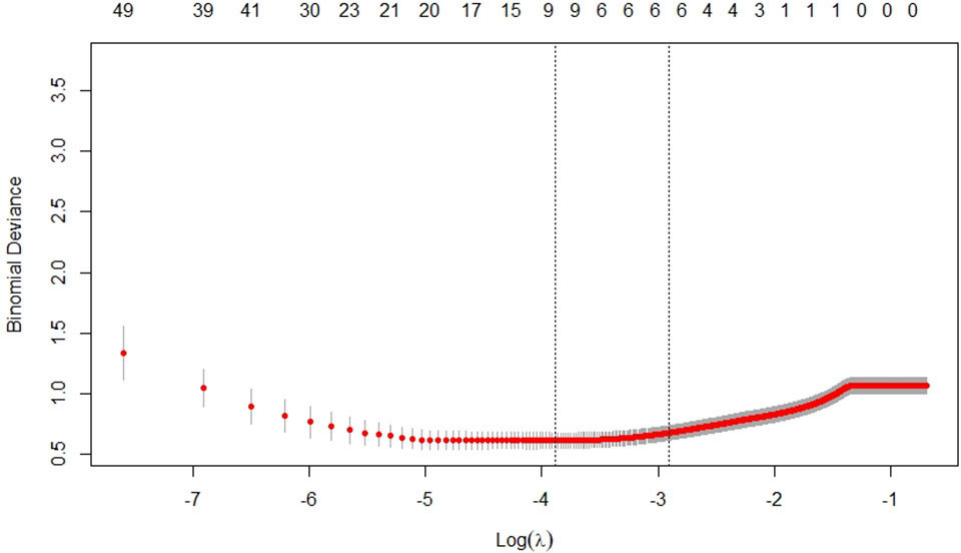
Selection of texture features by the least absolute shrinkage and selection operator (LASSO) regression in the training cohort.


Based on the training cohort, we extracted 279 texture features for each ROI. According to the result of reproducibility analysis by two ultrasound doctors, 250 radiomic features had good consistency (ICC ≥ 0.75). Through the LASSO regression (**Figure 3**), the following six optimal variables were selected: Skewness, Horzl_RLNonUni, Horzl_GLevNonU, WavEnLL_s.3, WavEnLH_s.3, and WavEnLH_s.4. Based on these six features, the radiomics score was calculated using the following formula:

$$Radiomics\;score\;=\;-3.675163\;-\;Skewness\;\times \;2.24776\;\times \;10^{-1}$$

$$-\;Horzl\_RLNonUni\;\times \;9.498166\;\times \;10^{-6}$$

$$-\;Horzl\_GLevNonU\;\times \;3.05807\;\times \;10^{-4}$$

$$+\;WavEnLL\_s.3\;\times \;6.213542\;\times \;10^{-5}$$

$$+\;WavEnLH\_s.3\;\times \;6.650157\;\times \;10^{-3}$$

$$+\;WavEnLH\_s.4\;\times \;1.14583\;\times \;10^{-3}$$

### Development and validation of the model

In the training cohort, a univariate analysis was performed on 14 observation indexes (**Table 2**). A multivariate logistic regression was used to analyze the selected variables (*p*<0.05) to determine the independent risk factors for the model (**Table 2**). Based on radiomics score, BI-RADS and palpable mass as independent risk variables (*p*<0.05), the logistic regression model was established by the following function (**Table 3**):

**Table 2:** Results of the univariate and multivariate logistic regression analysis in the training cohort.

	Univariate logistic regression analysis		Multivariate logistic regression analysis	
	OR (95% CI)	*P*-value	OR (95% CI)	*P*-value
Age	0.97 (0.87-1.07)	0.559		
BI-RADS				
3-4A 4B-5	Ref. 79.78 (4.81-3889.12)	0.007	Ref. 54.42 (12.98-308.27)	<0.001
Tumor Size (cm)	2.87 (0.86-11.72)	0.100		
Duct Ectasia				
None Ectasia	Ref. 0.02 (0.00-1.19)	0.055		
Palpable Mass				
None Palpable	Ref. 68.03 (5.04-2661.21)	0.006	Ref. 22.89 (4.33-144.54)	<0.001
Echo Pattern				
Hypoechoic Complex Echo	Ref. 0.06 (0.00-36.89)	0.539		
Echo Distribution				
Uniform Non-Uniform	Ref. 13.98 (0.72-659.76)	0.112		
Boundary				
Well-Circumscribed Obscure	Ref. 7.85 (0.77-154.81)	0.109		
Shape				
Regular Irregular	Ref. 5.83 (0.18-215.56)	0.316		
Orientation				
Parallel Not Parallel	Ref. 0.67 (0.02-32.58)	0.819		
Posterior Feature				
None Attenuation	Ref. 0.48 (0.02-15.15)	0.642		
Calcification				
None Calcification	Ref. 0.14 (0.00-2.01)	0.184		
Vascularity Distribution				
Absent Internal	Ref. 8.28 (0.84-146.54)	0.088		
Radiomics Score	0.13 (0.02-0.54)	0.019	0.20 (0.07-0.46)	0.001

**Table 3:** Variable assignment table in the logistic regression model.

Variable	Code	Variable assignment
BI-RADS	X_1_	3-4A=0, 4B-5 = 1
Palpable Mass	X_2_	None=0, Palpable=1
Radiomics Score	X_3_	Score


Logit(P) = −5.880236 + 3.996762X1 + 3.130755X2 − 1.603437X3

**Figure 4:**
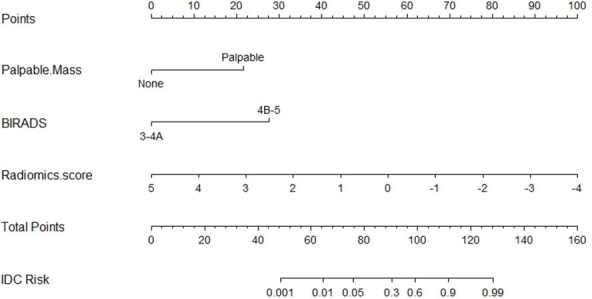
The nomogram was established based on the model.

The nomogram was developed based on the logistic regression model (**Figure 4**) (21, 22).

**Figure 5:**
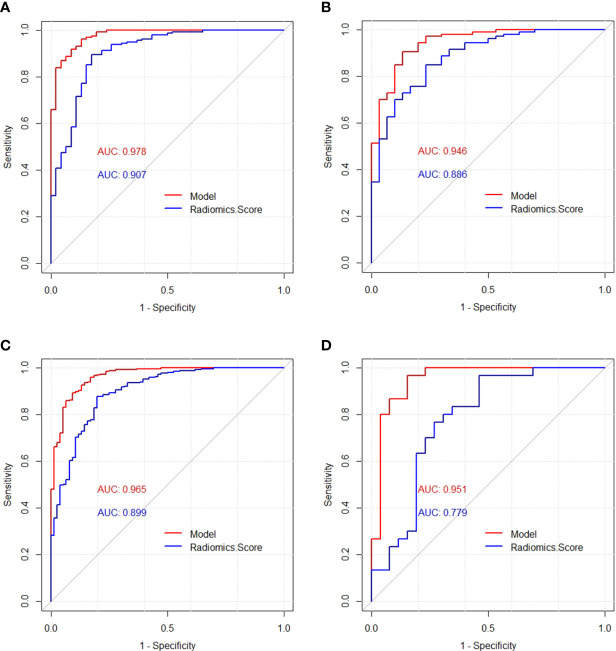
Receiver operating characteristic (ROC) curves of the model and radiomics score in the training cohort **(A)**, validation cohort **(B)**, total dataset **(C)** and independent validation cohort **(D)**, respectively.

The diagnostic performances of the model and the radiomics scores were verified by the ROC analysis (**Figure 5**). The AUC was used to quantify discrimination. In the training cohort, the AUC of the model and the radiomics score were 0.978 (95% confidence interval [CI: 0.960-0.997]) and 0.907 (95% confidence interval [CI: 0.854-0.960]), respectively. In the validation cohort, the AUC of the model and the radiomics score were 0.946 (95% confidence interval [CI: 0.903-0.990]) and 0.886 (95% confidence interval [CI: 0.821-0.951]), respectively. In the total dataset, the AUC of the model and the radiomics score were 0.965 (95% confidence interval [CI: 0.943-0.986]) and 0.899 (95% confidence interval [CI: 0.858-0.939]), respectively. In the independent validation cohort, the AUCs of the model and the radiomics score were 0.951 (95% confidence interval [CI: 0.891-1]) and 0.779 (95% confidence interval [CI: 0.650-0.909]), respectively. (**Table 4**) According to DeLong’s test, there were statistically significant differences (*p*<0.05) between the model and radiomics scores.

**Table 4:** AUCs of the radiomics score and model.

	Training cohort (n=208)	*P*-value	Validation cohort (n=137)	*P*-value	Total dataset (n=345)	*P*-value	Independent validation cohort (n=56)	*P*-value
	AUC (95% CI)		AUC (95% CI)		AUC (95% CI)		AUC (95% CI)	
Model	0.978 (0.960-0.997)		0.946 (0.903-0.990)		0.965 (0.943-0.986)		0.951 (0.891-1)	
Radiomics score	0.907 (0.854-0.960)		0.886 (0.821-0.951)		0.899 (0.858-0.939)		0.779 (0.650-0.909)	
Model *vs*. Radiomics score		0.005		0.025		<0.001		0.002

**Figure 6:**
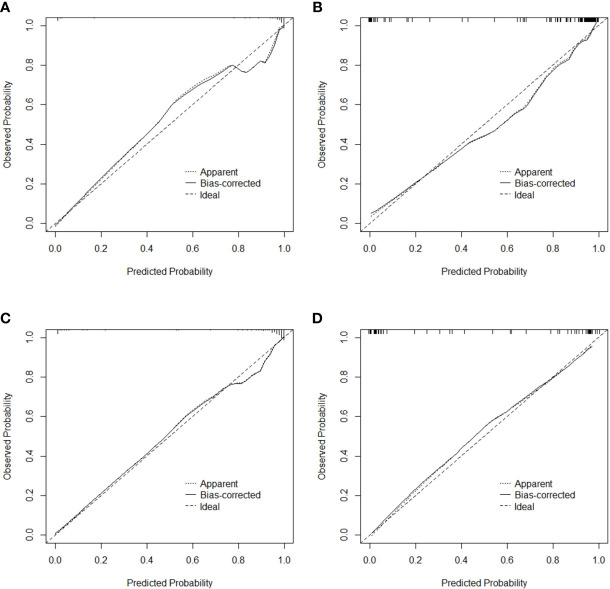
Calibration curve for the model in the training cohort **(A)**, validation cohort **(B)**, total dataset **(C)** and independent validation cohort **(D)**, respectively.

**Figure 7:**
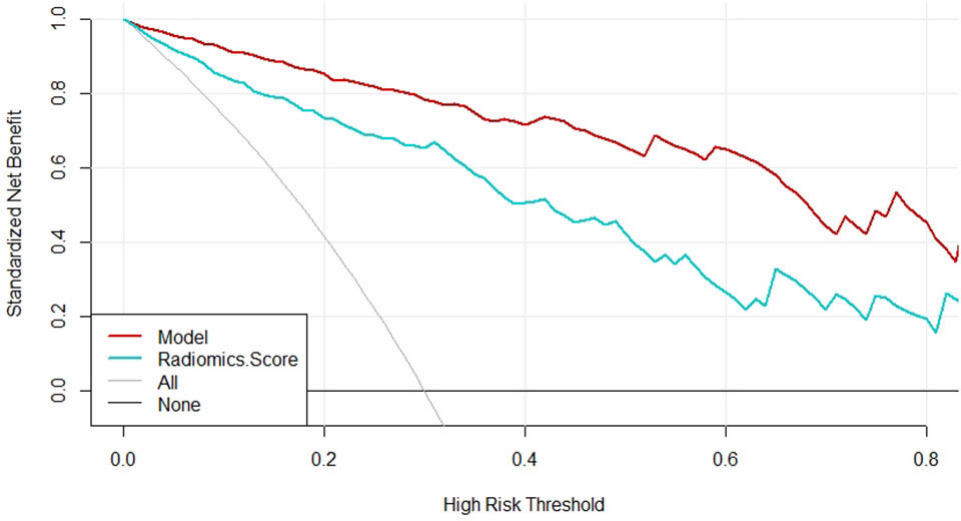
Decision curve analysis for the model and radiomics score.

The specificity, sensitivity, accuracy, Youden index, negative predictive value, positive predictive value, false positive rate, true positive rate, true negative rate and false negative rate of the model and the radiomics score in the training cohort, the validation cohort, the total dataset and in the independent validation cohort were shown in **Table 5**, respectively. The Brier score of 0.066 suggested a high accuracy of the model. The calibration curve demonstrated good agreement between the prediction and the pathological results (**Figure 6**). The DCA was plotted for the model (**Figure 7**). It demonstrated that if the threshold probability is more than 5%, using the model to predict SA and IDC will be more beneficial than either the treat-all-patients scheme (assuming all lesions are IDC) or the treat-none scheme (assuming all lesions are SA). Based on Leave-One-Out Cross-Validation, the Kappa value of this model was 0.79, which proved that the model had good stability.

**Table 5:** The evaluation index of the radiomics score and model.

	Training cohort (n=208)		Validation cohort (n=137)		Total dataset (n=345)		Independent validation cohort (n=56)	
	Model	Radiomics score	Model	Radiomics score	Model	Radiomics score	Model	Radiomics score
**specificity**	0.913	0.826	0.867	0.767	0.908	0.803	0.846	0.538
**sensitivity**	0.920	0.895	0.907	0.850	0.892	0.877	0.967	0.967
**accuracy**	0.918	0.880	0.898	0.832	0.896	0.861	0.911	0.768
**Youden index**	0.833	0.721	0.774	0.617	0.800	0.680	0.813	0.505
**npv**	0.764	0.691	0.722	0.590	0.704	0.649	0.957	0.933
**ppv**	0.974	0.948	0.960	0.929	0.972	0.940	0.879	0.707
**fpr**	0.087	0.174	0.133	0.233	0.092	0.197	0.154	0.462
**tpr**	0.920	0.895	0.907	0.850	0.892	0.877	0.967	0.967
**tnr**	0.913	0.826	0.867	0.767	0.908	0.803	0.846	0.538
**fnr**	0.080	0.105	0.093	0.150	0.108	0.123	0.033	0.033

(npv,negative predictive value; ppv,positive predictive value; fpr,false positive rate; tpr,true positive rate; tnr,true negative rate; fnr,false negative rate).

According to the model, the lower the radiomics score, the higher the BI-RADS classification, the more palpable the mass, and the greater the possibility of IDC.

## Discussion

We developed and validated an ultrasound-based radiomics model, which included the radiomics score, BI-RADS and palpable mass, to distinguish between SA and IDC. Although the radiomics score we created was proved to have a high AUC value, the model showed a better diagnostic efficacy and clinical utility than the radiomics score alone, which indicates the superiority of the model in disease identification.

SA is an IDC-mimicking benign proliferative breast lesion, which is usually asymptomatic or only palpated with a mass. In previous studies, it has been confirmed that SA can imitate IDC clinically, radiologically, and pathologically, so it is necessary to distinguish between SA and IDC (1–5, 7). As a convenient, affordable, and radiation-free imaging examination, the US is a most widely used breast screening technique. Liu et al. found that US BI-RADS atlas and elastography are powerful tools in diagnosing SA (1). Shao et al. asserted that an enhanced US could improve the diagnostic accuracy of SA (23). However, these researchers used a subjective analysis or expensive inspections. The texture analysis is a new computer-aided technology used for quantitative analyses of image information through algorithms, which can prevent the subjectivity of ultrasonic examinations and BI-RADS classifications (10, 11). To our knowledge, no research has focused on ultrasonic omics to distinguish between SA and IDC using a texture analysis.

We selected six radiomic features based on a regression analysis, including one histogram parameter (Skewness), two grey level run-length matrix (RLM) parameters (Horzl_RLNonUni and Horzl_GLevNonU), and three Haar wavelet transform parameters (WavEnLL_s.3, WavEnLH_s.3, and WavEnLH_s.4). The texture analysis was normalized by MaZda software. According to the coefficients, Skewness, Horzl_RLNonUni, and Horzl_GLevNonU were negatively correlated with the radiomics score. That is, the larger the Skewness, Horzl_RLNonUni, and Horzl_GLevNonU, the lower the radiomics score and the higher the probability of IDC. In addition, the three Haar wavelet transform parameters were all positively correlated with the radiomics score, which indicates that when these three parameters are larger, the radiomics score is higher and the probability of IDC is lower. Furthermore, skewness seemed to contribute most to the radiomics score.

The histogram is computed based on the intensity of the pixels without considering any spatial relations between the pixels within the image (12). As one characteristic variable of a histogram, a high skewness means an asymmetrical distribution with a long right tail. A tumor with a high skewness of signal intensity is mainly composed of fibrosis or stroma. In this study, skewness was positively correlated with the malignant degree of the tumor, which may be related to the high gray intensity of the image caused by hyperplasia, fibrosis, calcification, and tumor cell accumulation in the IDC glands. Previous studies have shown that a high mammographic density independently predicts the risk of breast cancer and that a high skewness of a tumor might be related to poor survival (24–26). Our observations were consistent with these previous reports. On a gray-level image, the RLM quantifies the coarseness of a texture in a specific direction. When runs are equally distributed throughout the gray levels, the function of gray-level non-uniformity reaches its lowest values. If the runs are equally distributed throughout the lengths, the function of run length non-uniformity has a low value (27). In our study, Horzl_RLNonUni and Horzl_GLevNonU were negatively correlated with the radiomics score, which meant that the gray levels and the lengths of IDC were nonuniform. This is consistent with our observation of the IDC ultrasonic features. The wavelet transform provides time/space and frequency (or scale) resolution information of the signal/image and the details of the image at different frequencies, which reflects the detailed features of the image. When the image is clearer or the frequency is richer, the parameter value is higher. The Haar wavelet has mainly been used for the feature extraction of breast cancer diagnoses in many studies (28).In this study, the selected three Haar wavelet transform parameters were all positively correlated with the radiomics score, which meant that the IDC texture images were blurred. This may be due to the heterogeneity of IDC cells and the proliferation of tumor blood vessels, which are prone to necrosis and make the tumor image blurry.

Despite the promising performance of the radiomics score, the model of our study, which combined ultrasonic characteristics, BI-RADS, clinical information, and radiomic features, had the advantages of being affordable and objective, suggesting that it is beneficial to combine a texture analysis with ultrasonic features and clinical manifestations in future medical work. Based on the univariate logistic regression, each index was gradually fitted, and three characteristics were screened out as indicators to distinguish between SA and IDC. Soo-Yeon Kim et al. proposed that BI-RADS 4B or 5 was independently related to malignant tumors, and had a high upgrade rate (29). Based on our findings, BI-RADS 3 or 4A suggests that SA is possible, and a higher classification tends to be malignant. A palpable mass with a lower radiomics score further suggests IDC. The results were basically consistent with previous research conclusions (1, 29, 30). In addition, based on the multivariate logistic regression analysis, the influence of confounding factors was eliminated, and the final three variables were obtained, including the radiomics score, BI-RADS and palpable mass, which were used as independent influence factors and were selected to develop the model.

There are some limitations of the current study that need to be further investigated. (1) This study was a retrospective analysis, therefore it was difficult to completely overcome the operator dependency of the initial examination, making a bias error inevitable. (2) This study was a single-center research study, so the number of SA and IDC cases was limited. The performance of this model needs to be verified by other centers and a larger cohort in the future. (3) We only included patients with SA and IDC, though the differences in the texture features for the pathological subtypes of breast cancer and adenosis can be analyzed in the future.

## Conclusion

The model in our study based on radiomics, ultrasonic features, and clinical manifestations can be used to distinguish SA from IDC, which showed good stability and diagnostic performance. The model can be considered a potential candidate diagnostic tool for breast lesions and can contribute to effective clinical diagnosis and treatment.

## Data availability statement

The original contributions presented in the study are included in the article/Supplementary Material. Further inquiries can be directed to the corresponding author.

## Ethics statement

The study was conducted in accordance with the principles of the Declaration of Helsinki, and the study protocol was approved by the First Affiliated Hospital of Guangxi Medical University ethics committee. The study is of the retrospective nature, patient consent for inclusion was waived.

## Author contributions

QH wrote the main manuscript text. WN and XT prepared tables and figures. YG was fully responsible for the design of this study and data processing. All authors contributed to the article and approved the submitted version.
